# Denitrification and Biodiversity of Denitrifiers in a High-Mountain Mediterranean Lake

**DOI:** 10.3389/fmicb.2017.01911

**Published:** 2017-10-06

**Authors:** Antonio Castellano-Hinojosa, David Correa-Galeote, Presentación Carrillo, Eulogio J. Bedmar, Juan M. Medina-Sánchez

**Affiliations:** ^1^Departamento de Microbiología del Suelo y Sistemas Simbióticos, Estación Experimental del Zaidín, Consejo Superior de Investigaciones Científicas, Granada, Spain; ^2^Instituto Universitario de Investigación del Agua, Universidad de Granada, Granada, Spain; ^3^Departamento de Ecología, Facultad de Ciencias, Universidad de Granada, Granada, Spain

**Keywords:** high-mountain lake, nitrate, nitrous oxide, water column, sediments, 16S rRNA gene, *nosZ* gene, biodiversity

## Abstract

Wet deposition of reactive nitrogen (Nr) species is considered a main factor contributing to N inputs, of which nitrate (${NO}_{3}^{-}$) is usually the major component in high-mountain lakes. The microbial group of denitrifiers are largely responsible for reduction of nitrate to molecular dinitrogen (N_2_) in terrestrial and aquatic ecosystems, but the role of denitrification in removal of contaminant nitrates in high-mountain lakes is not well understood. We have used the oligotrophic, high-altitude La Caldera lake in the Sierra Nevada range (Spain) as a model to study the role of denitrification in nitrate removal. Dissolved inorganic Nr concentration in the water column of la Caldera, mainly nitrate, decreased over the ice-free season which was not associated with growth of microbial plankton or variations in the ultraviolet radiation. Denitrification activity, estimated as nitrous oxide (N_2_O) production, was measured in the water column and in sediments of the lake, and had maximal values in the month of August. Relative abundance of denitrifying bacteria in sediments was studied by quantitative polymerase chain reaction of the 16S rRNA and the two phylogenetically distinct clades *nosZ*I and *nosZ*II genes encoding nitrous oxide reductases. Diversity of denitrifiers in sediments was assessed using a culture-dependent approach and after the construction of clone libraries employing the *nosZ*I gene as a molecular marker. In addition to genera *Polymorphum*, *Paracoccus*, *Azospirillum*, *Pseudomonas*, *Hyphomicrobium*, *Thauera*, and *Methylophaga*, which were present in the clone libraries, *Arthrobacter*, *Burkholderia*, and *Rhizobium* were also detected in culture media that were not found in the clone libraries. Analysis of biological activities involved in the C, N, P, and S cycles from sediments revealed that nitrate was not a limiting nutrient in the lake, allowed N_2_O production and determined denitrifiers’ community structure. All these results indicate that denitrification could be a major biochemical process responsible for the N losses that occur in La Caldera lake.

## Introduction

In the context of global alterations of the biogeochemical cycles, inland aquatic ecosystems are considered as true sentinels of change due to its high connectivity with terrestrial ecosystems (Williamson et al., 2009). Among them, high-mountain lakes located in remote areas at high altitude, with small catchment areas and small volume of water, are strongly influenced by processes of atmospheric transport (Brahney et al., 2015; Cabrerizo et al., 2017). These features, and the fact that their food webs are usually simple, but with complex interactions (Carrillo et al., 2006), make them excellent witnesses of global change and suitable model ecosystems to quantify basic processes related to the functioning and alterations of biogeochemical cycles (Catalán et al., 2006; Medina-Sánchez et al., 2013; Durán et al., 2016).

That of N is one of the biogeochemical cycles under greater alteration currently at global scale (Galloway et al., 2008). This acceleration is due to the increase in the availability of reactive nitrogen (Nr) species as a result of human activity, including industrially produced fertilizer, combustion of fossil fuels, transportation, biomass and agricultural burning, intensification of biological N_2_ fixation by agriculture, etc. (Fowler et al., 2013; Hertel et al., 2014). Due to alterations of the natural N cycle, human health, ecosystem integrity, resilience, and biodiversity are already suffering serious and potentially irreversible effects (Erisman et al., 2011, 2013; Shibata et al., 2015). The long-term consequences are yet unknown, but the current human impact on this cycle has exceeded the operating limits considered safe (Rockström et al., 2009; Steffen et al., 2015).

Within the N cycle, dissimilatory reduction of nitrate to ammonium, chemoautotrophic denitrification via sulfur or iron oxidation, anammox and, mainly denitrification are the only known biological processes involved in removal of Nr species in the biosphere (Burgin and Hamilton, 2007; Galloway et al., 2013; Erisman et al., 2015). During denitrification, nitrate (${NO}_{3}^{-}$) can be reduced to N_2_ via the formation of nitrite (NO_2_^-^), nitric oxide (NO) and nitrous oxide (N_2_O) under oxygen-limiting conditions. Those reactions are carried out by the nitrate- (Nar/Nap), nitrite- (nirK/NirS), NO- (cNor/qNor), and nitrous oxide-reductase (NosZI and NosZII) enzymes encoded by the *narG*/*napA*, *nirK*/*nirS*, c-*norC*/q-*norC*, and *nosZ* clades I (*nosZ*I) and II (*nosZ*II) genes, respectively (Zumft, 1997; van Spanning et al., 2007; Richardson, 2011; Bedmar et al., 2013). Many denitrifiers do not have, or do not express, the complete set of enzymes required for denitrification, which, in turn, may result not only in nitrite formation, but also in the release into the atmosphere of NO and N_2_O, two potent greenhouse gases that are involved in global climatic change, formation of acid rain and destruction of the ozone layer (Ravishankara et al., 2009; IPCC, 2013; Erisman et al., 2015).

Flow of Nr species through the atmosphere causes their deposition on aquatic ecosystems, including those in remote areas such as high-mountain lakes (Catalán, 1992; Catalán et al., 1993; Elser et al., 2009a,b; Gąsiorowski and Sienkiewicz, 2013). Through validated chemical transport models, high wet depositions of Nr have been reported in North and Northeastern Spain, decreasing along the NE–SW axis of the Iberian Peninsula (García-Gómez et al., 2014). In Sierra Nevada mountains (southern Spain), inputs of N are mainly related to wet deposition, which are not related to Saharan dust export toward the Mediterranean region (Morales-Baquero et al., 2006; Morales-Baquero and Pérez-Martínez, 2016). Long-term observations in the high-mountain lake La Caldera within the Sierra Nevada range shows that, despite Nr depositions, an inter-annually seasonal trend of decreasing dissolved inorganic nitrogen (DIN) can be established, with nitrate (${NO}_{3}^{-}$) as the predominant inorganic form (Carrillo et al., 1996; Medina-Sánchez et al., 1999; Delgado-Molina et al., 2009). Decreases in DIN, however, were not associated with the dynamics of total biomass of La Caldera, so that reasons for N losses remain elusive.

Incubation experiments with ^15^N-nitrite revealed N loss occurring in the chemocline through denitrification in the high-mountain, meromictic Cadagno lake (Switzerland) (Halm et al., 2009). Also, a growing gradient of DIN toward the hypolimnetic redox transition zone indicated the existence of sulfide-dependent denitrifiers in the water column of the meromictic south-alpine Lugano lake (Wenk et al., 2013). Moreover, a high bacterial diversity, including anaerobic microorganisms and the presence of denitrifying bacteria in epilithic biofilms was shown in oligotrophic mountain lakes (Bartrons et al., 2012), despite these microhabitats had been considered unsuitable for denitrification (Vila-Costa et al., 2014). Previous studies have reported the dominance of alpha and beta proteobacteria in the water column of La Caldera lake (Reboleiro-Rivas et al., 2013), but denitrifiers have not been determined in this ecosystem, so that whether or not denitrification is involved in nitrate removal in the oligotrophic lake of La Caldera is not known. Accordingly, the main goal of this study was to analyze denitrification activity in the water column and in sediments that could be responsible for the seasonal N loss pattern found in La Caldera lake over an interannual temporal scale. For this purpose denitrification activity was examined as N_2_O production in the water column and sediment samples. Abundance of denitrifiers was estimated by quantitative polymerase chain reaction (qPCR) and bacterial identification was made after construction of clone libraries with *nosZ* genes. Finally, abiotic factors involved in control of denitrification activity were also determined.

## Materials and Methods

### Study Site

La Caldera is an oligotrophic, high-mountain lake located on the southern slope of Sierra Nevada range (Southeast Spain), at 3050 masl, very close to the level of summits (36°55′–37°15′N, 2°31′–3°40′W). With a catchment area of ∼1.46 km^2^, it stands at a glacial cirque of rocky siliceous nature (shales, quartzites), tectonically belonging to the Mulhacén mantle of the Nevado-Filábride complex of Betic ranges. The lake has an area of ∼0.02 km^2^, an average depth of 4.3 m, and lacks visible tributaries or effluents and littoral vegetation.

### Sampling, Physicochemical and Biological Variables of the Water Column

Based on a consistent seasonal pattern at interannual long-term scale of nitrate dynamics and planktonic community development along the ice-free period (Carrillo et al., 1996, 2008; Villar-Argaiz et al., 2001; Delgado-Molina et al., 2009), sampling was performed during the ice-free period on June 9, August 1, and October 13, 2015. At the maximum depth point of the lake, temperature, dissolved oxygen (DO), conductivity, total dissolved solids (TDS), and pH were measured *in situ* along the water column using a multiparameter probe (TuroWater Quality Analysis T-611, Sandy Bay, TAS, Australia). Vertical profiles of solar radiation in the water column were determined at noon with a BIC compact four-channel radiometer (Biospherical Instruments, CA, United States) equipped with three channels in the ultraviolet region (UVR) of the spectra (305, 320, and 380 nm) and one broadband channel for photosynthetic active radiation (PAR; 400–700 nm). Diffuse attenuation coefficients for downward irradiance (*K_d_*) were calculated from the slope of the linear regression of natural logarithm of downwelling irradiance vs. depth for each wavelength range considered. A large sample size (pairs irradiance-depth data, *n* > 100) was used and a good fit (*R*^2^ > 0.90) was found for all regressions. Lake water samples were collected with an acid-cleaned horizontal 6 L van Dorn sampler at the deepest central station from three to four depths depending on the water column size and date (0.5 m below surface, 0.5 m above bottom, and two intermediate depths, **Table 1**). Total phosphorus (TP), total dissolved phosphorus (TDP), total nitrogen (TN), soluble reactive phosphorous (SRP), nitrate (${NO}_{3}^{-}$), dissolved organic carbon (DOC), and chlorophyll *a* (Chl *a*) in the water samples were analyzed as described previously (Carrillo et al., 2015) following standard methods (APHA, 2005). Bacterial abundance in the water column was quantified by the flow cytometry technique (FACSCanto, Becton Dickinson Biosciences) as described in Dorado-García et al. (2014). Microbial plankton biomass (MPB) in La Caldera lake is composed mainly of nanoplanktonic algae and heterotrophic bacteria, because other components of the microbial loop such as ciliates, heterotrophic nanoflagellates, and autotrophic picoplankton are not detected (Dorado-García et al., 2014; Cabrerizo et al., 2017). Hence, MBP was estimated as: MPB = AB + BB, where AB is the algal biomass calculated by multiplying a C:Chl *a* ratio of 7.48 (obtained from Carrillo et al., 2015) by the measured Chl *a*; BB is the bacterial biomass calculated as the product of the carbon content per bacterial cell (obtained from Carrillo et al., 2015) by the measured bacterial abundance.

**Table 1:** Physicochemical and biological properties of the water column from La Caldera lake (Sierra Nevada, Spain).

Sampling month	Depth (m)	TP (μg P L^-1^)	TDP (μg P L^-1^)	SRP (μg P L^-1^)	TN (mg N L^-1^)	${NO}_{3}^{-}$ (mg N L^-1^)	DOC (mg L^-1^)	MPB (μg C L^-1^)
June	0.5	2.4	1.2	0.12	0.30	0.32	0.40	21.34
	2	2.6	1.6	0.14	0.35	0.31	0.42	21.10
	4	3.1	1.8	0.23	0.50	0.32	0.43	21.19
	6	3.1	4.1	0.20	0.46	0.38	0.50	24.71
August	0.5	4.2	1.2	0.60	0.22	0.11	0.41	45.09
	2	5.7	2.8	0.15	0.23	0.10	0.37	31.84
	4	5.8	2.8	0.14	0.25	0.09	0.32	42.63
	6	5.8	2.3	0.54	0.13	0.12	0.31	43.00
October	0.5	4.5	1.9	0.32	0.10	0.06	0.95	19.13
	1.5	6.2	2.5	0.32	0.12	0.06	0.87	19.30
	3	7.0	2.8	1.17	0.15	0.07	0.61	19.70


*Samples were taken on June 9, August 1, and October 13. TP, total phosphorus; TDP, total dissolved phosphorus; SRP, soluble reactive phosphorus; TN, total nitrogen; NO_*3*_^-^, nitrate content; DOC, dissolved organic carbon; MPB, microbial plankton biomass.*

Sediment samples were taken from the top layer (0–10 cm) at four sampling sites (Supplementary Figure S1). They were kept refrigerated, transported to the laboratory and maintained at -80°C until use.

### Enzymatic Analysis

Raw sediment and water column samples were used to analyze enzymatic activities (EA) related to C, N, P, and S biogeochemical cycles. Sediment moisture was determined gravimetrically by oven-drying the samples at 105°C for 24 h. Dehydrogenase was used to estimate overall microbial activity, β-glucosidase (GLU) as the enzyme that catalyses the hydrolysis of disaccharides (C cycle), urease (UR) which catalyses the hydrolysis of urea to CO_2_ and NH_3_ (N cycle), acid phosphatase (AP) for estimation of the enzymes responsible for the hydrolysis of phosphate esters (P cycle), and arylsulphatase (AS) as a measure of the enzymes catalyzing the hydrolysis of organic sulfate esters (S cycle). Dehydrogenase was assayed according to García et al. (1997), GLU, AP, and AS were estimated according to Tabatabai (1982), and UR activity was determined as indicated by Kandeler and Gerber (1988). Briefly, these techniques were based on a controlled incubation of the samples after adding the initial substrate (INT, 2-*p*-iodophenyl-3-*p*-nitrophenyl-5-tetrazolium for dehydrogenase; *p*NG, 4-nitrophenyl-β-D-glucopyranoside for GLU; urea for UR activity; *p*NPP, 4-nitrophenyl phosphate for AP; and *p*NPS, *p*-nitrophenyl sulfate for AS, respectively) and measuring the ending product of each enzyme reaction colorimetrically (INTF, iodonitrotetrazolium formazan for dehydrogenase; *p*NP, *p*-nitrophenol for GLU, AP, and AS; and NH_4_^+^ for UR activity).

EA ratios (GLU:AP, GLU:UR, GLU:AS, UR:AP, UR:AS) were calculated as indicators of nutrient-ratio limitations (C:P, C:N, C:S, N;P, N:S, respectively) according to previous studies on EA (Sinsabaugh et al., 2009; Sinsabaugh and Follstad-Shah, 2012; Velasco-Ayuso et al., 2017). Propagation errors were used to calculate the variance of each EA ratio.

### Determination of N_2_O and N_2_

N_2_O production by the water column (N_2_O*_W_*) and sediment (N_2_O*_S_*) samples was assayed as indicated earlier (Tortosa et al., 2011). Briefly, 25 g of sediments (covered with a 2 cm layer of water from the lake) or 25 mL of water from the water columns were placed in 125 mL glass bottles and closed with serum rubber caps to allow injection and withdrawal of gas samples. Then, 10% of the internal atmosphere of the flasks was removed and acetylene was injected to inhibit nitrous oxide reductase activity (Yoshinari and Knowles, 1976). After incubation at temperatures measured in the sediment and water column, samples (0.5 mL) were withdrawn from the headspace of the flasks and injected into an HP 4890D gas chromatograph equipped with an electron capture detector and a Porapak Q 80/100 mesh packed column. N_2_ was the carrier gas at 28 mL min^-1^ flow rate and the injector, column and detector temperatures were 125, 60, and 375°C, respectively. Denitrification activity was calculated from the N_2_O increases during incubation using the Bunsen coefficient for the N_2_O dissolved in water. In parallel experiments, N_2_O production was also determined after incubation of the samples without acetylene. N_2_ production was estimated as the difference between N_2_O emission with and without acetylene. N_2_O concentrations were calculated using 2% (v/v) N_2_O standard (Air Liquid).

### DNA Extraction from Sediments and Quantification of N Cycle-Associated Microbial Community

DNA was extracted from thawed 250 mg sediment samples using the PowerSoil^®^ DNA isolation kit according to the manufacturer’s instructions. Quality and size of soil DNA were checked by electrophoresis on 1% agarose. DNA was also quantified by spectrophotometry at 260 nm using a BioPhotometer (Eppendorf, Hamburg, Germany). The size of the denitrifier community was estimated by qPCR of *nosZ* gene clades I (Henry et al., 2006) and II (Jones et al., 2012) fragments as described earlier (Correa-Galeote et al., 2013). Primers were used to amplify a 267 bp fragment of *nosZ* within clade I and 720 bp fragment of *nosZ* within clade II. The total bacterial community was quantified using 16S rRNA gene as molecular marker as described by López-Gutiérrez et al. (2004). Reactions were carried out in an ABI Prism 7900 Sequence Detection System (Applied Biosystems). Quantification was based on the fluorescence intensity of the SYBR Green dye during amplification. qPCR assays were performed in triplicate for each of the four samples taken in each sampling date. Standard curves were obtained using serial dilutions of linearized plasmids containing cloned *nosZ* clades I and II and 16S rRNA genes amplified from bacterial strains. PCR efficiency for the different assays ranged between 90 and 99%. No template controls gave null or negligible values. Presence of PCR inhibitors in DNA extracted from soil was estimated by (i) diluting soil DNA extract and (ii) mixing a known amount of standard DNA to soil DNA extract prior to qPCR. In all cases, inhibition was not detected. Methodological evaluation of the real-time PCR assays showed a good reproducibility of 95.0 ± 12% between two runs.

### Clone Libraries Construction and DNA Sequencing

*nosZ* genes amplicons of clades I and II obtained during PCR assays for libraries construction were purified using the QIAquick PCR purification kit (Qiagen, Germany) and cloned using the pGEM-T Easy cloning kit according to the manufacturer’s instructions (Promega, United States). The recombinant *Escherichia coli* JM109 cells were inoculated onto solid Luria Bertani medium (Miller, 1972) containing ampicillin and X-Gal (5-bromo-4-chloro-3-indolyl-β-D-galactopyranoside), and grown overnight at 37°C. White colonies were screened by PCR using the vector primers Sp6 and T7 (Invitrogen). Purity of amplified products was checked by observation of a unique band of the expected size in a 1% agarose gel stained with GelRed as indicated by the manufacturer’s (Biotium Inc., United States). Nucleotide sequences of clones containing inserts of the expected size were determined by sequencing with the vector primer Sp6 and the BigDye terminator cycle kit v3.1 (Applied Biosystems, United States) according to the manufacturer’s instructions, followed by electrophoresis on an ABI 3100 genetic analyzer (Applied Biosystems, United States) at the sequencing facilities of Estación Experimental del Zaidín, CSIC, Granada, Spain. Three clone libraries, JCL, ACL, and OCL, were obtained that contained *nosZ* genes from sediments of La Caldera lake taken in June, August, and October, respectively.

### Phylogenetic Analysis and Diversity Indexes

The DNA sequences of *nosZ* gene fragments were aligned by using the ClustalW program available in the Geneious software package (version 6.0.3, Biomatters, New Zealand). Vector sequence was removed and discrepancies in alignment verified manually. The obtained sequences were compared against database sequences using the BLASTN program in Geneious and those showing similarity higher than 95% of those previously deposited in GenBank for *nosZ* were selected as positives. A phylogenetic tree was constructed from a matrix of pairwise genetic distances by using the neighbor-joining method available in MEGA 7 (Kumar et al., 2015). Bootstrap analysis was based on 1000 resamplings. The Good’s coverage values and the Shannon–Wiener and Simpson indexes were calculated using PAST software (v3.14).

### Isolation of Denitrifying Bacteria from Sediments and Culture Conditions

For each sampling time, sediment samples (250 mg) were placed in microtubes containing 1 mL sterile saline solution, shaken in a vortex for 1 min, and centrifuged at 6000 rpm for 1 min in a microfuge. Then, 1 mL aliquots of the supernatant were taken and 1:100, 1:250, and 1:500 dilutions (v/v) were used to inoculate Petri dishes containing either solid G2M11, G3M12, or G4M3 media (Heylen et al., 2006), and placed inside anaerobic Anaerocult^®^ jars (Millipore). The atmosphere inside the jar was made anoxic by using the commercial Anaerocult^®^ system (Millipore). Cultures were incubated at 30°C until the appearance of colony forming units (CFUs). They were further selected by microscopic observation with a microscope Nikon CFI60 so that they represented all the different colony types appeared on the plates. Tryptone soya agar medium was used for routine bacterial growth.

### DNA Extraction and PCR Amplification of 16S rRNA Gene from Bacterial Isolates

Genomic DNA was isolated from bacterial cells as previously described (El Batanony et al., 2015). The quantity of DNA was determined by using a Nanodrop spectrophotometer (NanoDrop ND1000). To cluster the isolates, repetitive extragenic palindromic-PCR (REP-PCR) were performed using primers REPIR-I and REP2-I (de Bruijn, 1992). PCR amplifications of 16S rRNA gene fragments were carried out using the two opposing primers fD1 and rD1 (Herrera-Cervera et al., 1999). Amplification products were purified using the Qiagen PCR product purification system and subjected to cycle sequencing using the same primers as for PCR amplification, with ABI Prism dye chemistry and analyzed with a 3130 xl automatic sequencer at the sequencing facilities of the Estación Experimental del Zaidín, CSIC, Granada, Spain. The 16S rRNA gene sequences were compared to those deposited in EzTaxon-e (Kim et al., 2012).

### Accession Numbers

Sequences have been deposited in GenBank with accession numbers LT900218–LT900337.

### Statistical Analyses

Significant differences among dates for each biological variable were tested by paired *t*-tests. To assess if the decrease in ${NO}_{3}^{-}$ content over the ice-free period was related to N-uptake due to the growth of the microbial plankton in water column, a regression analysis (MPB vs. ${NO}_{3}^{-}$) was performed. The seasonal distribution patterns of the independent abiotic (temperature, pH, DO, TN, TP, TDP, SRP, N_2_O_W_, N_2_O_S_, ${NO}_{3}^{-}$, and DOC) and dependent biotic (total abundance of 16S rRNA and *nosZ* clades I and II genes) variables in lake La Caldera were studied by ordinations using non-parametric multidimensional scaling (NMDS), aided by the Primer software (PRIMER-E, vs. 6.0, Plymouth, United Kingdom). The data sets were transformed to log (*x* + 1) and normalized. Sample-resemblance matrices were generated using the Bray–Curtis similarity coefficient. Values of stress level on the NMDS plots <0.2 indicate that they give an appropriate representation of the data distribution (Clarke and Warwick, 2001; Clarke et al., 2008). Spearman rank correlations of each variable were calculated and are represented in the plots as vectors that illustrate their directional influence and role in the ordination. To find potential relations between the biotic data and the environmental variables, a BIO-ENV analysis was performed. Global permutation tests (499 permutations, which is the maximum under the given number of permutations) were conducted to determine the highest value obtained which is called the BEST value. This value designates which subset of the abiotic variables best explains the distribution of the biotic data between samples. Stepwise multiple regression analysis was performed to assess the relative strength of the best abiotic variables selected in BEST for each biotic-dependent variable. Statistical analyses were done using the package Statgraphics (Statistical Graphics Corporation).

## Results

### Physicochemical Properties

Main physicochemical and biological characteristics determined in the water column of La Caldera lake are shown in **Table 1** and Supplementary Figure S2. Temperature of the water column exhibited a slight inverse stratification in June, and a slight thermal stratification in August and October. Except for October, the water column was nearly oxygen-saturated for all depths and sampling dates. Mean pH values for the water column were low in June (6.8), and reached values above neutrality in August (8.5) and October (8.03). TP and TDP values increased with depth and over the ice-free season. DOC values were <1 mg L^-1^ and peaked in October (0.81 mg L^-1^). TN decreased over the ice-free season and, as expected, ${NO}_{3}^{-}$ concentration followed a similar pattern with values varying from 0.33 mg N L^-1^ in June to 0.1 mg N L^-1^ in August and 0.063 mg N L^-1^ in October. The dynamic of ${NO}_{3}^{-}$ over the ice-free season did not explained the variance of MPB in the water column (*r*^2^ = 0.06, *p* > 0.45) indicating that this seasonal change was not associated with growth of microbial plankton. Attenuation coefficients (*K_d_*) for UVR and PAR were lower in August than in June, whereas their highest values were found in October (Supplementary Figure S2). TDS increased hardly through the ice-free season and values of conductivity remained without measurable variations over the ice-free period (data not shown).

### Enzymatic Activities

**FIGURE 1:**
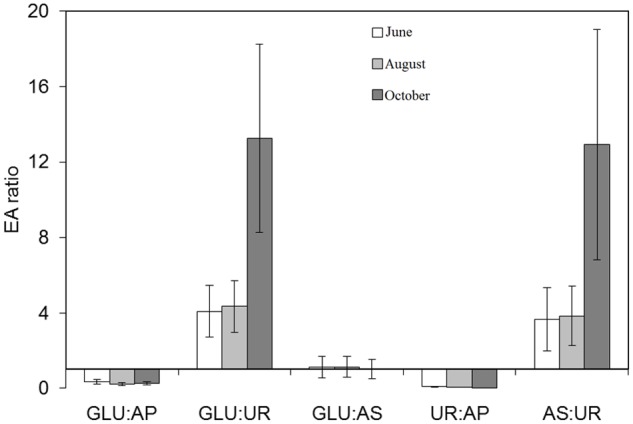
Enzymatic activities (EA) ratios analyzed in sediment samples taken along the ice-free season on June 9, August 1, and October 13, 2015 in lake La Caldera (Sierra Nevada, Spain). GLU, β-glucosidase; UR, urease; AP, acid phosphatase, AS, arylsulphatase. *X*-axis is the threshold value = 1. Error bars are calculated by propagation errors.

EA in sediments varied with the sampling times (Supplementary Table S1). Dehydrogenase, β-glucosidase, phosphatase, and arylsulfatase increased between June and August and decreased in October, with the lowest activity after thawing. In contrast, UR showed the lowest value in October. Despite repeated attempts to analyze EA in raw samples of the water column of the lake, obtained values were below the detection limits. The EA ratios varied from values below 1 (GLU:AP, UR:AP), not distinct from 1 (GLU:AS), to above 1 (GLU:UR, AS:UR), and this general pattern was kept or accentuated over the ice-free period (**Figure 1**).

### N_2_O and N_2_ Emissions

Production of N_2_O was detected in both sediment and water column samples of La Caldera lake (**Table 2**). Mean emissions of N_2_O diminished over the ice-free season. When acetylene was included to inhibit nitrous oxide reductase activity, production of N_2_O increased 5.2-, 4.7-, and 1.2-fold in sediments and 1.2-, 1.4-, and 1.4-fold in the water column from June to October. N_2_ emissions decreased following a similar pattern to that of N_2_O and values ranged from 538.9 to 7.4 (pmol N_2_ g^-1^ day^-1^) in sediments between June and October and from 38.9 to 16.9 (pmol N_2_ g^-1^ day^-1^) in the water column.

**Table 2:** Production of nitrous oxide (pmol N_2_O g^-1^ day^-1^) in water column and sediments from La Caldera lake (Sierra Nevada, Spain).

	Sampling month					
	
Treatment	June		August		October	
			
	Sediment	Water	Sediment	Water	Sediment	Water
Without acetylene	128.3 ± 32.2c	198.4 ± 39.3c	62.1 ± 17.1b	71.4 ± 19.5b	31.7 ± 11.4a	41.2 ± 11.9a
With acetylene	667.2 ± 55.1d	237.3 ± 39.7c	289.5 ± 40.4c	96.4 ± 22.1b	39.1 ± 13.1a	58.1 ± 13.7a


*Samples were taken on June 9, August 1, and October 13, 2015. Sediments were incubated in the absence and in the presence of acetylene. For each row different letters indicate significant differences according to paired *t*-tests (*p* ≤ 0.05, *n* = 5).*

### Abundance of 16S rRNA and *nosZ* Genes

The average number of copies of the 16S rRNA and *nosZ* clades I and II genes are shown in **Table 3**. The copy number of the 16S rRNA gene per nanogram of DNA in June was significantly lower than those estimated in August and October Abundance of the *nosZ* clade I gene was greater in August, followed by that found in October, and the lowest abundance in June. Differences in abundance of the *nosZ* clade II gene were not detected in samples taken in June and October, which were lower than those estimated in August. This month showed the highest relative abundance of denitrifiers as revealed by the *nosZ* (I + II)/16S rRNA ratio in comparison with those estimated in June and October (**Table 3**). At all three sampling dates, percentage of denitrifiers containing the *nosZ*I gene was higher than those with *nosZ*II. In addition, clades I and II of *nosZ* genes had different seasonal pattern as members of clade II decreased over the ice-free period and those of clade I peaked at August.

**Table 3:** Relative abundance of 16S rRNA and clade I and II *nosZ* genes in sediments from La Caldera lake (Sierra Nevada, Spain).

	Sampling month		
	
	June	August	October
16S rRNA	1.61 × 10^5^a	5.05 × 10^6^b	2.90 × 10^6^b
*nosZ*I	2.91 × 10^2^a	5.68 × 10^4^c	1.79 × 10^3^b
*nosZ*II	2.31 × 10^2^a	5.57 × 10^3^b	1.69 × 10^2^a
*nosZ*I/16S rRNA	0.18b	1.12c	0.06a
*nosZ*II/16S rRNA	0.14b	0.11b	0.01a
*nosZ*I + *nosZ*II/16S rRNA)	0.32b	1.24c	0.07a


*Samples were taken on June 9, August 1, and October 13. Values are expressed as the number of copies of the gene per nanogram of DNA. For each row different letters indicate significant differences according to paired *t*-tests (*p* ≤ 0.05, *n* = 4).*

### Analysis of Clone Libraries: Coverage and Diversity Indexes

**FIGURE 2:**
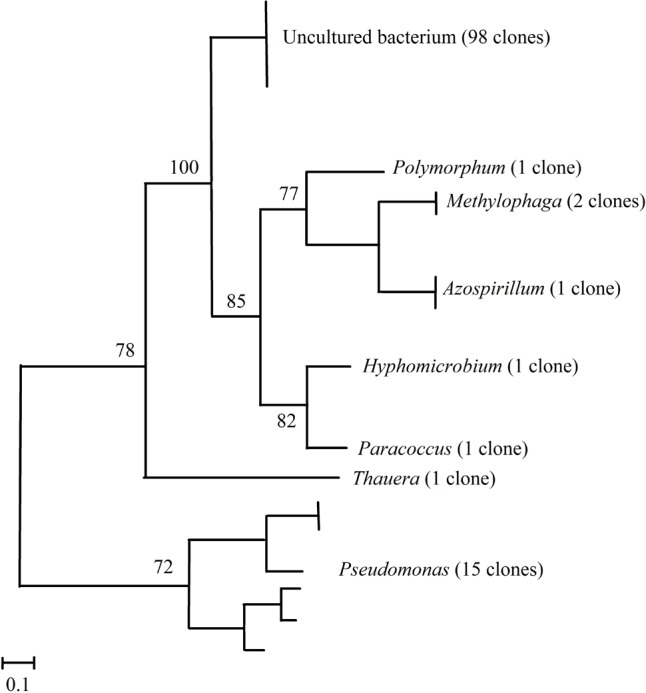
Neighbor-joining phylogenetic tree based on partial *nosZ* sequences of clones isolated from sediments of La Caldera lake (Sierra Nevada, Spain). The analysis was based on 250 nucleotides. Bootstraps values are indicated as percentages derived from 1000 replications. Values lower than 70 are not shown. Bar, 0.1 nucleotide substitution per 100 nucleotides.

Three clone libraries containing each 40 *nosZ* clade I genes were obtained corresponding to sediment samples taken in June, August, and October, respectively. Construction of a phylogenetic tree based on the 120 *nosZ* sequences showed they distributed into eight clusters corresponding to uncultured bacteria and to genera *Polymorphum*, *Paracoccus*, *Azospirillum*, *Pseudomonas*, *Hyphomicrobium*, *Thauera*, and *Methylophaga* (**Figure 2**). Out of the 120 clones analyzed, members of the uncultured bacteria were the most abundant (81.66%) followed by those of the classes Gammaproteobacteria (14.1%), Alphaproteobacteria (3.3%), and Betaproteobacteria (0.8%). The Good’s coverage index for each library was higher than 80%, which indicates that the sampling effort was enough to permit extrapolations for analysis of total *nosZ* gene biodiversity in the sediment samples. The diversity indices showed quite similar values between June and October genomic libraries (Shannon–Wiener index: 0.57–0.61, Simpson index: 0.27–0.27), and were higher for the August library (Shannon–Wiener index: 0.64, Simpson index: 0.39). Uncultured bacteria were the most abundant in the three libraries (34, 30, and 34 clones in June, August, and October, respectively). *Pseudomonas* was also present in the three libraries with three, nine, and three clones in June, August, and October, respectively. Genus *Methylophaga* was represented by two clones in the August library and the remaining genera were represented by one clone each.

### Linking Abundance of Targeted Genes with Environmental Variables in La Caldera Lake

**FIGURE 3:**
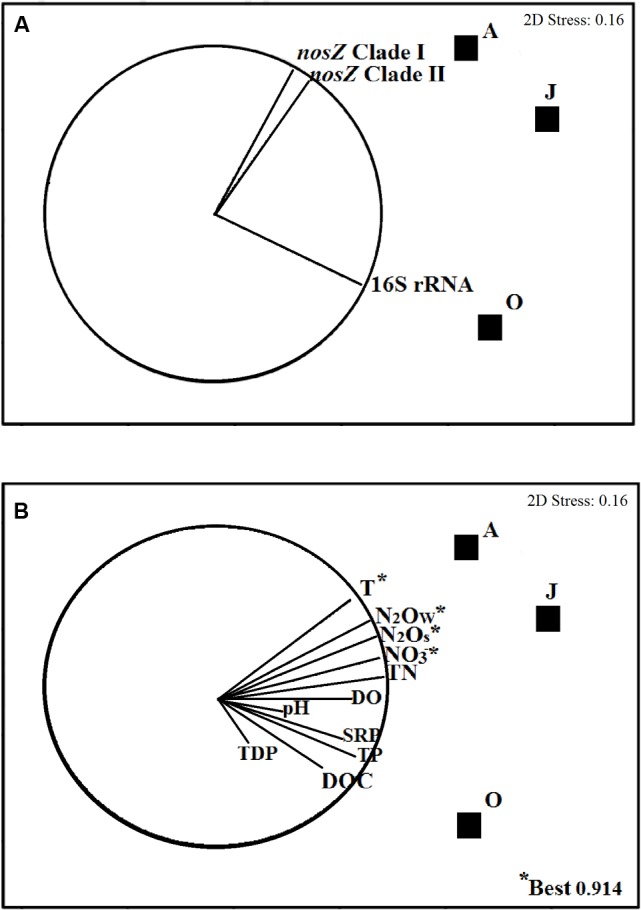
Non-parametric multidimensional scaling (NMDS) plots illustrating ordinations of sediments samples from La Caldera lake (Sierra Nevada, Spain) over the ice-free season based on the abundance of copies of bacterial 16S rRNA and *nosZ* genes. Vectors in plots illustrate the direction and strength of the relationships between biotic **(A)** and abiotic variables **(B)**. Sampling months are represented by squares (June; August; October). The variables which best explained the distributions of the biological data according to BIO-ENV analysis are marked with an asterisk (^∗^). T, temperature; N_2_O*_w_*, production of N_2_O by water columns; N_2_O*_S_*, production of N_2_O by sediments; ${NO}_{3}^{-}$, nitrate content; TN, total nitrogen; DO, dissolved oxygen; SRP, reactive soluble phosphorus; TP, total phosphorus; DOC, dissolved organic carbon; TDP, total dissolve phosphorus. The N_2_O values used in plots are those obtained in samples that were incubated without acetylene.

An NMDS plot showed that *nosZ*I and *nosZ*II genes grouped together and separated from the 16S rRNA gene (**Figure 3A**). Relative abundance of the 16S rRNA gene correlated (*r* ≥ 0.50) with the variables (in decreasing order of strength) TDP, DOC, TP, SRP, and pH, whereas both the *nosZ* clades I and II genes correlated with the variables (in decreasing order of strength) temperature, N_2_O*_w_*, N_2_O*_S_*, ${NO}_{3}^{-}$, and TN (*r* ≥ 0.50) (**Figure 3B**). According to the global permutation test, the significance of all vectors was *p* < 0.01, which means that a sufficient number of data was used in the BIO-ENV analysis.

According to the BIO-ENV analysis, temperature, ${NO}_{3}^{-}$, N_2_O*_S_*, and N_2_O*_w_* were the best abiotic variables explaining (91.4%) the ordination of the samples based on the changes of the biotic data (**Figure 3B**). An additional stepwise multiple regression analysis (**Table 4**) shows that variance of the 16S rRNA gene abundance of La Caldera was explained mainly by temperature (31%) and ${NO}_{3}^{-}$ (30%). However, the analysis also revealed that *nosZ* clades I and II gene abundances were explained firstly by ${NO}_{3}^{-}$ (55 and 52%, respectively), followed by N_2_O*_S_* (21 and 20%, respectively) and by N_2_O*_w_* (15 and 17%, respectively).

**Table 4:** Multiple stepwise regression analysis among biotic-dependent variables (targeted genes abundance) and the independent variables that best explained NMDS ordination.

Dependent variable	Independent variable	β	Multiple *R*^2^	*R*^2^ change	*p*
16S rRNA gene abundance	T	0.55	0.31	0.31	0.007
	${NO}_{3}^{-}$	0.51	0.61	0.30	0.008
	N_2_O*_W_*	0.23	0.72	0.11	0.018
	N_2_O*_S_*	0.21	0.82	0.10	0.035
Clade I *nosZ* gene abundance	${NO}_{3}^{-}$	0.80	0.55	0.55	0.001
	N_2_O*_S_*	0.33	0.76	0.21	0.011
	N_2_O*_W_*	0.26	0.91	0.15	0.022
	T	0.11	0.97	0.06	0.035
Clade II *nosZ* gene abundance	${NO}_{3}^{-}$	0.76	0.52	0.52	0.001
	N_2_O*_S_*	0.29	0.72	0.20	0.012
	N_2_O*_W_*	0.27	0.89	0.17	0.016
	T	0.18	0.97	0.08	0.037


*NO_*3*_^-^, nitrate content; N_*2*_O_*W*_, production of N_*2*_O by water columns; N_*2*_O_*S*_, production of N_*2*_O by sediments; T, temperature; β, standardized regression coefficient; multiple *R*^**2**^, coefficient of multiple determination; *R*^2^ change, change in multiple *R*^*2*^ caused by entering a new variable in a single step; *p* indicate the significant effect on the considered variable.*

### Isolation of Denitrifying Bacteria from Sediments

Preliminar experiments designed to know the best media, whether G2M11, G3M12, or G4M3 (Heylen et al., 2006), to be used for isolation of denitrifiers from the sediments showed that G3M12 allowed appearance of a greater number of CFUs and that those that appeared in the G2M11 and G4M3 media were also present in G3M12 (data not shown). When that medium was used, 122 CFUs were obtained in June, a value that increased in the month of August to 209 and decreased to 52 in October. After morphological analysis, 31, 25, and 25 CFUs corresponding to June, August, and October, respectively, were selected which clustered in 29 different groups when subjected to REP-PCR (**Table 5**). Partial sequences of the 16S rRNA gene from a representative strain of each REP group and pairwise alignments between globally aligned sequences showed they were closely related to members of genera *Paracoccus*, *Polymorphum*, *Pseudomonas*, *Arthrobacter*, *Hyphomicrobium*, *Azospirillum*, *Methylophaga*, *Thauera*, *Burkholderia*, and *Rhizobium* (**Table 5**), of which *Arthrobacter*, *Burkholderia*, and *Rhizobium* had not been detected in the clone libraries. The representative strains used in this study can be considered as true denitrifiers as, after identification, they all were capable of growing again in G3M12 medium.

**Table 5:** REP-PCR groups and taxonomic identification of representative strains isolated from sediments of La Caldera lake (Sierra Nevada, Spain) taken on June 9, August 1, and October 13, 2015.

REP-PCR group	Strains	Closest relative genus on basis of 16S rRNA gene	Similarity according to EzTaxon-e (%)
I	**J14**	*Paracoccus*	97
II	**A25**	*Polymorphum*	96
III	**JX3**	*Paracoccus*	97
IV	**A7**, J3	*Pseudomonas*	98
V	**O13**	*Pseudomonas*	99
VI	**J7**	*Arthrobacter*	99
VII	**O12**, O11, O8, **O10**, O9, O3, **O5**, O2, O4	*Pseudomonas*	99
VIII	**A14**	*Hyphomicrobium*	97
IX	**A18**, O1	*Pseudomonas*	98
X	**O7**, O6, J6	*Pseudomonas*	99
XI	**J1**, J4	*Azospirillum*	97
XII	**O15**	*Pseudomonas*	97
XIII	**A3**, A4, J23	*Pseudomonas*	88
XIV	**JX4**, O20	*Pseudomonas*	97
XV	**A19**, A16	*Methylophaga*	97
XVI	**JX6**, O17	*Pseudomonas*	97
XVII	**O19**	*Pseudomonas*	96
XVIII	**O14**, O16	*Thauera*	97
IXX	**J21**, A1, A10, **A11**, A12, A17	*Pseudomonas*	99
XX	**J19**, J22, J25	*Paracoccus*	97
XXI	**J18**, JX1	*Paracoccus*	96
XXII	**J12**	*Burkholderia*	92
XXIII	**A2**	*Rhizobium*	100
XIV	**A20**, A21, A23	*Methylophaga*	99
XXV	**O22**, J8, O23	*Pseudomonas*	97
XXVI	**O25**, O24, O21	*Methylophaga*	100


*Strains in bold were chosen as the representative of each REP-PCR group.*

## Discussion

Despite increases in global Nr deposition, previous studies have consistently observed a regional pattern of decreasing DIN in recent decades in the Pyrenean lakes (Camarero and Catalán, 2012) as well as an inter-annually seasonal trend of decreasing DIN in the Sierra Nevada lakes, as La Caldera (Carrillo et al., 1996; Medina-Sánchez et al., 1999; Delgado-Molina et al., 2009). The latter trend can be also elicited from data reported in other studies on high altitude mountain lakes (Tiberti et al., 2013; Porcal et al., 2014; Santolaria et al., 2015). While P deposition can play a role in these trends (Camarero and Catalán, 2012), the present study extends those findings by showing it is likely the loss of N could be due to denitrification activity. As expected, ${NO}_{3}^{-}$ concentration decreased over the sampling dates and this was the predominant N form found in the lake (**Table 1**). Although UVR radiation has been involved in nitrate removal by photoreduction or photolysis (Brezonik and Fulkerson-Brekken, 1998; Porcal et al., 2014), *K_d_* and DOC variations over the ice-free period did not match those of ${NO}_{3}^{-}$ (**Table 1** and Supplementary Figure S2). Thus, it is unlikely that UV radiation is a main factor involved in nitrate removal in this ecosystem. On the other hand, our results indicate that decreases in ${NO}_{3}^{-}$ content were not associated with N-uptake due to the growth of the microbial plankton. This is in agreement with previous results which show that DIN in La Caldera lake did not relate to MPB or growth during the ice-free season (Villar-Argaiz et al., 2001; Carrillo et al., 2002; Medina-Sánchez et al., 2004; Dorado-García et al., 2014; Durán et al., 2016). In fact, N was not the main limiting nutrient for microbial plankton growth in the lake as indicated by the EA related to C, N, P, and S cycles. Given the link between the microbial metabolism and environmental energy/resource availability provided by the ratios between EA (Sinsabaugh et al., 2009; Sinsabaugh and Follstad-Shah, 2012), the calculated ratios in our study (GLU:AP < 1; GLU:UR > 1; GLU:AS ∼ 1, UR:AP < 1; AS:UR > 1) indicate a resource limitation ordered as P > C = S > N, an order which was even intensified over the ice-free period (**Figure 1**). This is consistent with the patterns of resource limitation found in La Caldera under events of Saharan dust-aerosol deposition (Velasco-Ayuso et al., 2017).

Despite the relatively low nitrate concentration, denitrification activity, measured as N_2_O production, was detected both in the water column and in the sediments (**Table 2**). On average, the presence of acetylene during assays for denitrification increased N_2_O production by 61 and 38% in the sediments and in the water column, respectively (**Table 2**), which points to the presence of a bacterial community with the capacity to carry out the complete denitrification process and, therefore, the ability to produce N_2_ as a final denitrification product. It is unlikely that nitrifiers substantially contributed to N_2_O production in La Caldera as the content of ammonium in the water column and sediments was negligible (data not shown). Relative contribution of sediment vs. water column to denitrification is difficult to estimate due to the inherent strong variations of size of both compartments intra- and inter-annually.

Assays based on the presence of the *nosZ* genes (clades I and II), or gene products, which catalyzes the reduction of N_2_O to N_2_, have successfully been used to relate community structure to denitrification (Throbäck et al., 2004; Peralta et al., 2010; Deslippe et al., 2014; Tatti et al., 2015; Barrett et al., 2016). Denitrifiers of both *nosZ*I and *nosZ*II clades were present in the sediments, and variations in their dynamics paralleled those of the total number of bacteria after determination by quantification of the 16S rRNA gene (**Table 3**). Because denitrifiers of clades I and II *nosZ* genes has also been shown in sediments of boreal lakes (Saarenheimo et al., 2015a), our study reinforces the relevance of studying *nosZ*I and *nosZ*II genes in aquatic ecosystems.

Diversity of bacterial denitrifiers in the lake was estimated by construction of clone libraries using the *nosZ* clades I and II genes as molecular markers. Altogether, genera found in the three clone libraries were *Polymorphum*, *Paracoccus*, *Azospirillum*, and *Hyphomicrobium* of the phylum Alphaproteobacteria, *Thauera* of the phylum Betaproteobacteria, *Pseudomonas* of the phylum Gammaproteobacteria, and *Methylophaga* of the Gammaproteobacteria, though 81.67% of the 120 sequences in the libraries corresponded to unclassified bacteria (**Figure 2**), which indicates the presence of hitherto uncultured bacterial groups in high-mountain lakes. Scala and Kerkhoff (2000) also found that uncultured bacteria were predominant when analyzing denitrifying communities in marine sediments. Among the identified genera, *Pseudomonas* was the most abundant and was found in samples from the three sampling dates (**Figure 2** and Supplementary Table S2). Our data, however, contrast with those obtained by pyrosequencing which indicate that genera *Burkholderia*, *Azospirillum*, and *Ochrobactrum* were the core *nosZ*-OTUs found in boreal lakes (Saarenheimo et al., 2015b). Values of the Shannon–Wiener and Simpson indexes for the JCL and OCL clone libraries were similar, and lower than those for the ACL library, which indicates that biodiversity of denitrifying bacteria was higher in August, coinciding with the highest water temperature (**Table 1**). Thus, NMDS ordinations (**Figure 3**) revealed that temperature, ${NO}_{3}^{-}$, and N_2_O emissions showed strong positive correlation with the abundance of denitrifiers. Positive correlations between N availability and denitrification capability have also been shown in boreal lakes (Kortelainen et al., 2000; Rissanen et al., 2011, 2013; Saarenheimo et al., 2015a).

The culture-dependent approach used in this study to isolate denitrifying bacteria from the lake La Caldera resulted in obtaining a total of 383 CFUs that grouped in 26 REP clusters. REP-PCR fingerprinting has been extensively used to cluster bacteria at the subspecies of strain level (de Bruijn, 1992) and is known to be a powerful tool for studies on microbial ecology and evolution (Ishii and Sadowsky, 2009). Interestingly, genera *Arthrobacter*, *Burkholderia*, and *Rhizobium* were identified that had not been detected in the clone libraries. This suggests that both culture-dependent and -independent methods are complementary to study bacterial biodiversity.

## Conclusion

**FIGURE 4:**
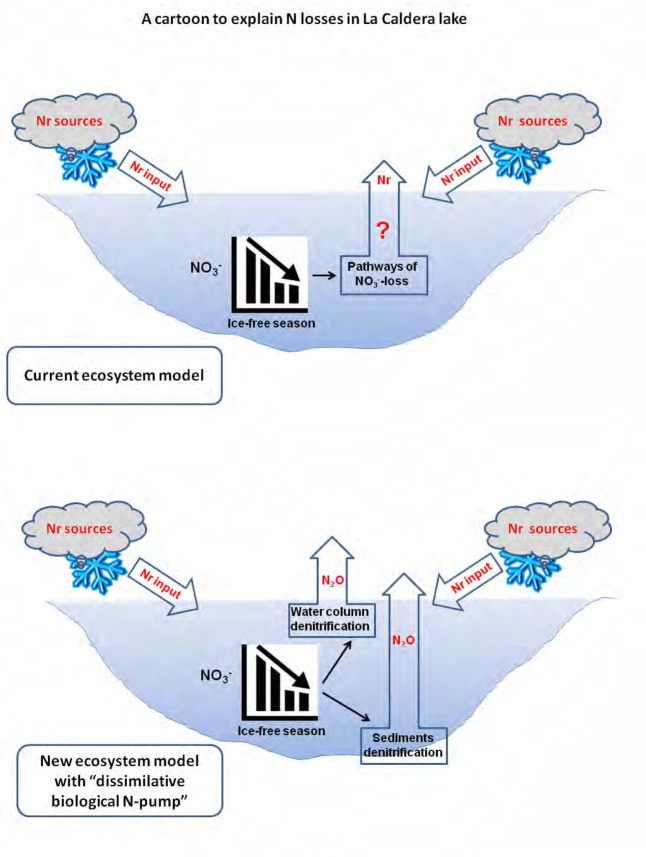
A conceptual model of how a “dissimilative biological N-pump” could explain the seasonal loss of N in La Caldera lake (Sierra Nevada, Spain).

Taken together, our results provide evidence for a noteworthy role of bacterial denitrification as a “dissimilative biological N-pump” in the water column and sediments that shapes the N cycle and explain the decreasing ${NO}_{3}^{-}$ content over the ice-free period in oligotrophic high-mountain lakes as La Caldera (**Figure 4**). In this study, environmental conditions, particularly the non-limiting N content, allowed N_2_O production and determined denitrifiers’ community structure. Therefore, this study provides evidence that denitrification can act as an auto-depurative mechanism for removal of contaminant nitrates in high-mountains lakes. Moreover, in other types of lakes where nitrate loads are higher, and non-limiting for plankton growth, denitrification is expected to be enhanced, which requires further research.

## Author Contributions

EB and JM-S conceived and designed the experiments. AC-H, DC-G, and JM-S performed the experiments. EB, PC, JM-S, and AC-H analyzed the data. EB, PC, and JM-S contributed reagents/materials/analysis tools. EB, JM-S, PC, and AC-H wrote the paper.

## Conflict of Interest Statement

The authors declare that the research was conducted in the absence of any commercial or financial relationships that could be construed as a potential conflict of interest.
