# Supplementary material for: Denitrification and Biodiversity of Denitrifiers in a High-Mountain Mediterranean Lake
Source: Front Microbiol. 2017 Oct 6;8:1911. doi: 10.3389/fmicb.2017.01911 (PMC5635049; doi:10.3389/fmicb.2017.01911)
Supplement: Supplementary file 1 [file Table_1.DOC]

TABLE S1 | Enzymatic activities in sediments from La Caldera lake (Sierra Nevada, Spain) taken in June 9^th^, August 1^st^ and October 13^th^ 2015.

|  | Sampling month | | |
| --- | --- | --- | --- |
|  | June | August | October |
| Dehydrogenase (µmol INTF g^-1^ h^-1^) | 12.7 | 22.2 | 16.9 |
| β-glucosidase  (µmol *p*NP g^-1^ h^-1^) | 14.7 | 19.6 | 15.9 |
| Urease  (µmol NH_4_^+^ g^-1^ h^-1^) | 3.6 | 4.5 | 1.2 |
| Acid phosphatase (µmol *p*NP g^-1^ h^-1^) | 44.8 | 82.5 | 65.4 |
| Arylsulphatase (µmol *p*NP g^-1^ h^-1^) | 13.2 | 17.3 | 15.5 |
