# Supplementary material for: Denitrification and Biodiversity of Denitrifiers in a High-Mountain Mediterranean Lake
Source: Front Microbiol. 2017 Oct 6;8:1911. doi: 10.3389/fmicb.2017.01911 (PMC5635049; doi:10.3389/fmicb.2017.01911)
Supplement: Supplementary file 2 [file Table_2.DOC]

TABLE S2 | Structure (genera) and abundance (number of clones) found in *nosZ* gene clone libraries from sediments of La Caldera lake (Sierra Nevada, Spain) taken in June 9^th^ (JCL), August 1^st^ (ACL) and October 13^th^ (OCL) 2015.

|  | Genomic library | | |
| --- | --- | --- | --- |
|  | JCL | ACL | OCL |
|  | Number of clones | | |
| Uncultured bacterium | 34 | 30 | 34 |
| *Pseudomonas* | 3 | 9 | 3 |
| *Methylophaga* | ND | ND | 2 |
| *Polymorphum* | 1 | ND | ND |
| *Paracoccus* | 1 | ND | ND |
| *Azospirillum* | 1 | ND | ND |
| *Hyphomicrobium* | ND | 1 | ND |
| *Thauera* | ND | ND | 1 |

ND: not detected
