# Supplementary material for: Denitrification and Biodiversity of Denitrifiers in a High-Mountain Mediterranean Lake
Source: Front Microbiol. 2017 Oct 6;8:1911. doi: 10.3389/fmicb.2017.01911 (PMC5635049; doi:10.3389/fmicb.2017.01911)
Supplement: Supplementary file 3 [file Presentation_1.PPTX]

## Slide 1
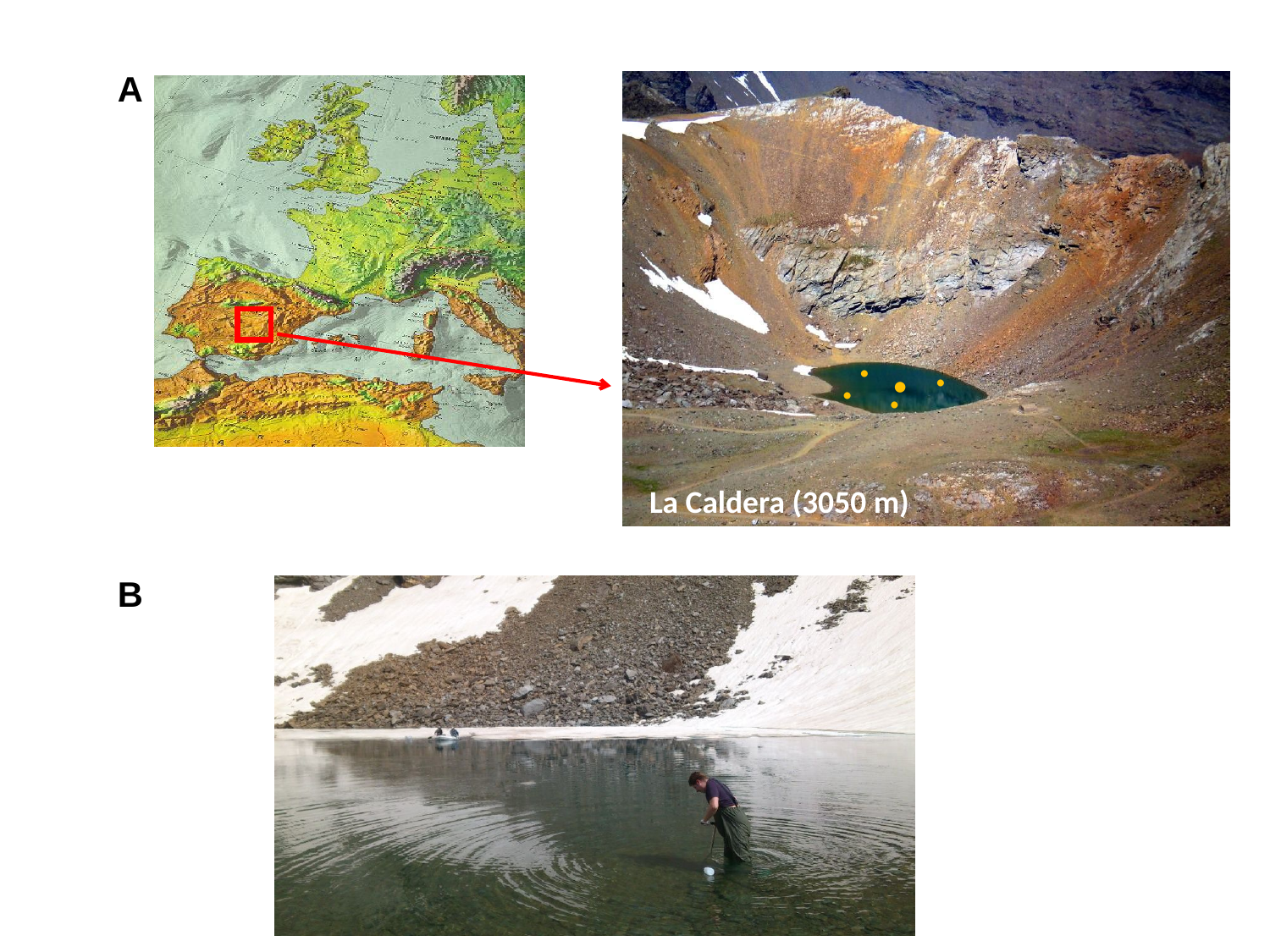

A
.
.
.
.
.
La Caldera (3050 m)
B

## Slide 2
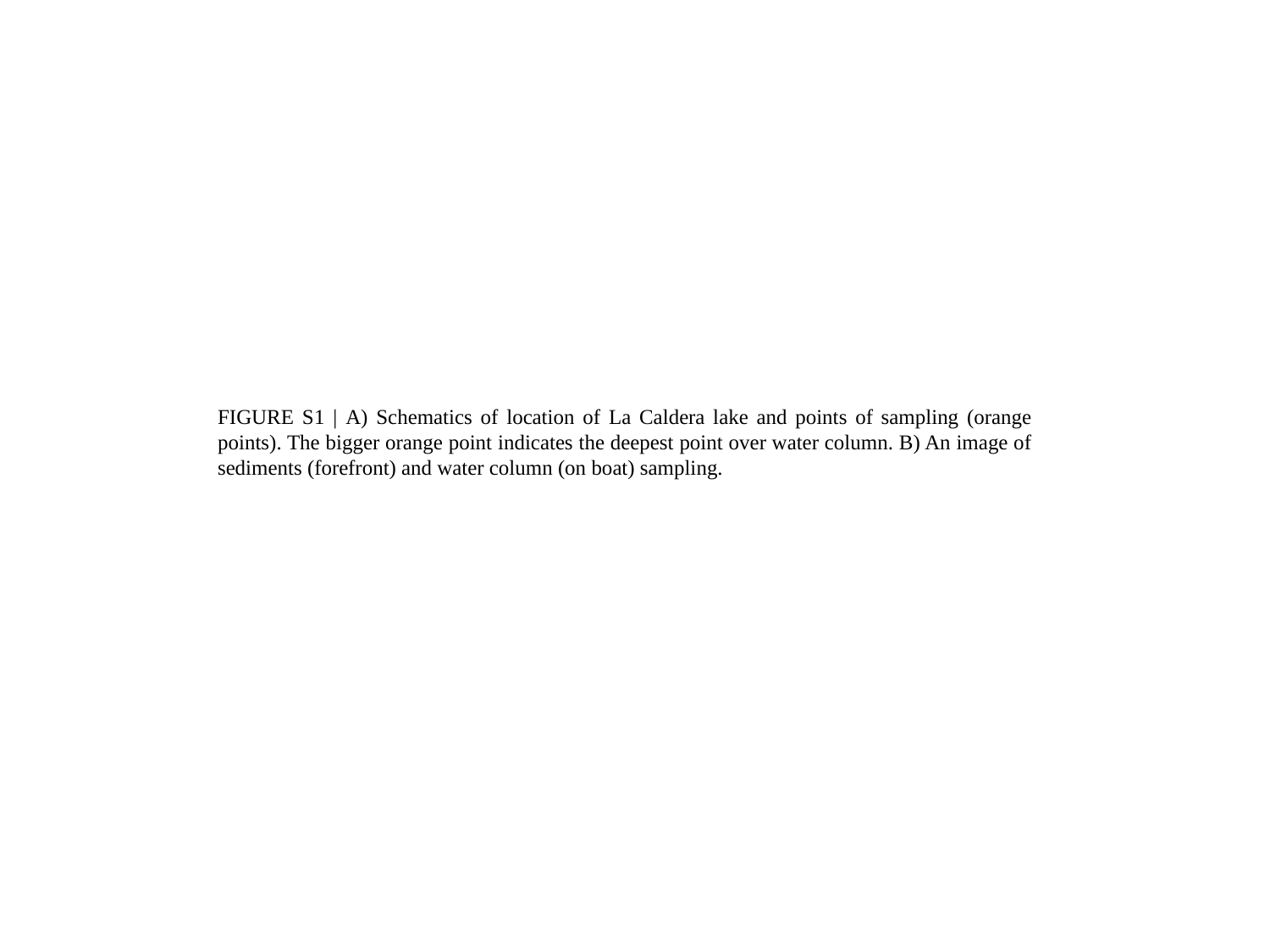

FIGURE S1 | A) Schematics of location of La Caldera lake and points of sampling (orange points). The bigger orange point indicates the deepest point over water column. B) An image of sediments (forefront) and water column (on boat) sampling.
