# Supplementary figures and images for: Denitrification and Biodiversity of Denitrifiers in a High-Mountain Mediterranean Lake
Source: Front Microbiol. 2017 Oct 6;8:1911. doi: 10.3389/fmicb.2017.01911 (PMC5635049; doi:10.3389/fmicb.2017.01911)

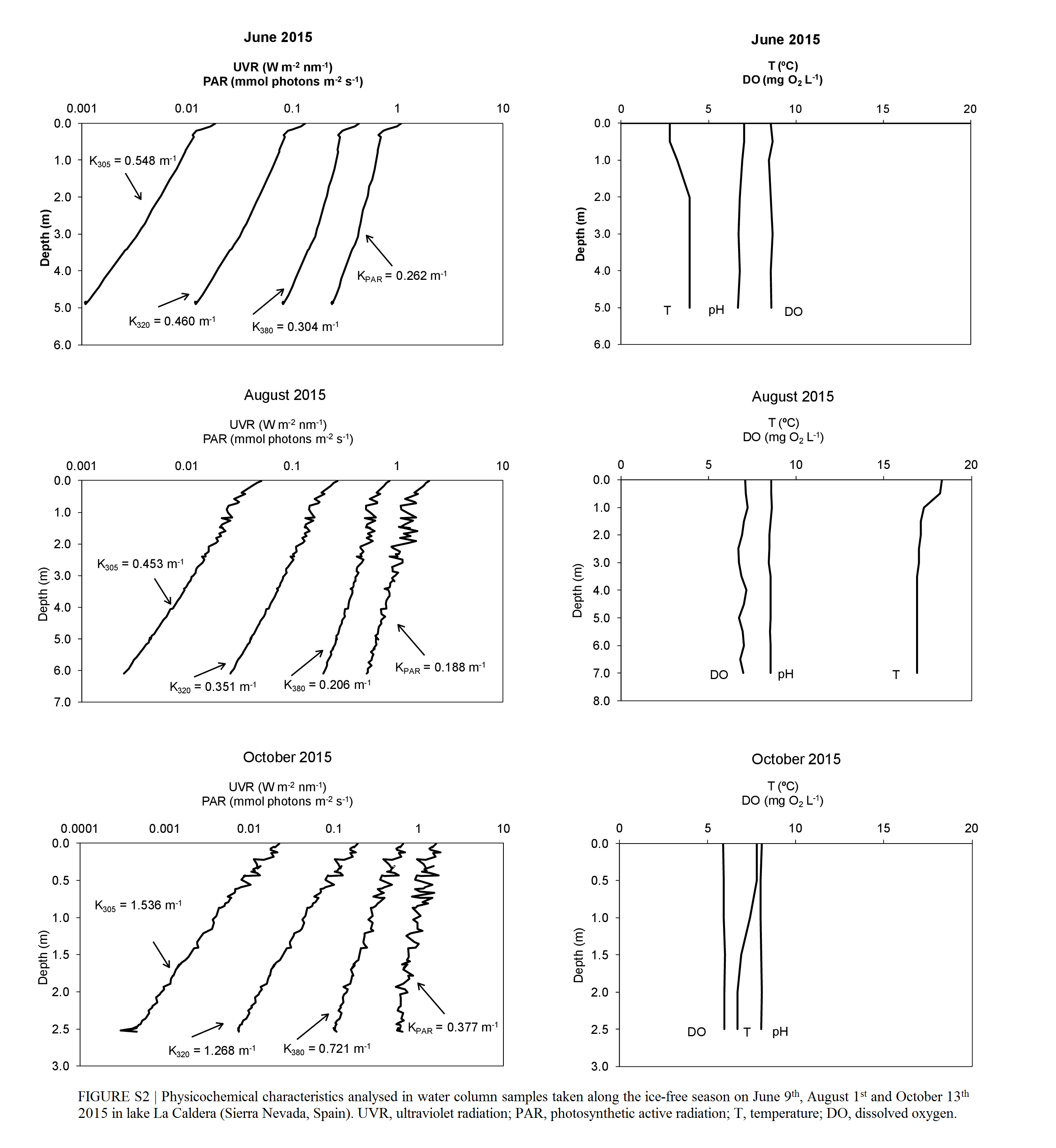

Supplement: Supplementary file 4 [file Image_2.TIFF]
